# *Fertilization Independent Endosperm* genes repress *NbGH3.6* and regulate the auxin level during shoot development in *Nicotiana benthamiana*

**DOI:** 10.1093/jxb/erw024

**Published:** 2016-02-11

**Authors:** Jun Zeng, Qi Ding, Hiroo Fukuda, Xin-Qiang He

**Affiliations:** ^1^State Key Laboratory of Protein and Plant Gene Research, College of Life Sciences, Peking University, Beijing 100871, China; ^2^Department of Biological Sciences, Graduate School of Science, The University of Tokyo, Bunkyo-ku, Tokyo 113-0033, Japan

**Keywords:** Auxin, FIE, GH3.6, *Nicotiana*, *benthamiana,* shoot development.

## Abstract

The *Fertilization Independent Endosperm* (*FIE*) gene is required to restrict endosperm development without fertilization, and it represses flowering during embryo and seedling development in *Arabidopsis thaliana*. However, the regulatory mechanism of the *FIE* gene in postembryonic shoot development is not well understood. Silencing of *Nicotiana benthamiana* homologues of the *FIE* gene, *NbFIE1* and *NbFIE2*, resulted in the enhanced outgrowth of axillary buds and the impairment of secondary xylem differentiation. RNA sequencing analysis found that one of the auxin-responsive *GRETCHEN HAGEN 3* (*GH3*) family genes, *NbGH3.6*, was upregulated and maintained a high expression during the time course of silencing *NbFIE* genes. Chromatin immunoprecipiation (ChIP)-PCR results showed a lack of H3K27me3 marks on *NbGH3.6* chromatin in *NbFIE*-silenced plants compared with negative control plants, indicating that *NbGH3.6* was a direct target of *NbFIE* genes during postembryonic shoot development. Moreover, the free IAA content was reduced significantly in *NbFIE*-silenced plants, which might cause the enhanced outgrowth of axillary buds as well as impaired secondary xylem differentiation. These results clearly indicated that *NbGH3.6* was a primary target of *NbFIE* genes during postembryonic shoot development, and *NbFIE* genes regulated axillary bud growth and secondary xylem formation through tuning endogenous auxin homeostasis, possibly by regulating the expression of the *NbGH3.6* gene.

## Highlight

*Fertilization Independent Endosperm* genes control shoot branching and secondary xylem differentiation in *Nicotiana benthamiana* by tuning auxin homeostasis, possibly by repressing the *NbGH3.6* gene through the histone methylation mark H3K27me3.

## Introduction

Higher plants display a variety of architectures that are defined by the degree of branching, internodal elongation, and shoot indeterminacy, which are mainly determined by meristem activity and hormone actions under the control of genetic and environmental programmes (Reinhardt and Kuhlemeier, 2002; Busov *et al.*, 2008; Wang and Li, 2008). Apical meristem indeterminacy and internodal elongation determine the height of mature plants. Branching of the shoot and inflorescence is the direct result of axillary meristem activity (Leyser, 2009; Wang *et al.*, 2014). The activity of cambium, a secondary meristem, results in secondary vascular tissue differentiation and increases of stem radial length, which also contribute to plant architecture (Larson, 1994).

Phytohormones are critical in the interactions between genetic and environmental programmes determining the creation of plant architecture. Among them auxin plays a vital role during this process (Chatfield *et al.*, 2000; Shimizu-Sato *et al.*, 2009; Müller and Leyser, 2011). Through the interaction with other hormones, like cytokinins and strigolactone, auxin regulates multiple plant growth and developmental processes, including the maintenance of apical dominance, leaf development, and vascular differentiation (McSteen, 2009; Shimizu-Sato *et al.*, 2009; Müller and Leyser, 2011).

Polycomb repressive complex 1 and 2 (PRC1 and 2) function as evolutionarily conserved transcription repressors by modulating chromatin modification (Schwartz and Pirrotta, 2007). The *Fertilization Independent Endosperm* (*FIE*) gene, encoding a WD40 homologue of *Drosophila* Extra Sex Comb (ESC), was first isolated as the causal gene of the mutation that caused autonomous endosperm proliferation and finally seed abortion in the absence of fertilization in *A. thaliana* (Ohad *et al.*, 1996; Chaudhury *et al.*, 1997; Ohad *et al.*, 1999). The FIE protein functions as a core structural component of the three putative PRC2 complexes, Fertilization Independent Seed (FIS), Embryonic Flower (EMF), and Vernalization (VRN), which function in seed development, vegetative phase transition and the vernalization response, respectively (Köhler and Villar, 2008; Hennig and Derkacheva, 2009). In *Arabidopsis* lines with suppression of the *FIE* gene, compared with wild-type plants, the number of the inflorescence stems increases while the inflorescence meristems cease activity with a few terminal flowers on stunted stems (Katz *et al.*, 2004). When the homologous gene of *FIE* is knocked out in *Physcomitrella patens*, the mutated moss displays overproduced buds that cannot accomplish the default development programme (Mosquna *et al.*, 2009). These results indicate that the *FIE* genes are involved in meristem activity. Our previous transcriptome analysis in *Populus* reveals that polycomb group genes, including homologues of the *SWINGER* (*SWN*), *MULTICOPY SUPRESSOR OF IRA1* (*MSI1*) and *FIE* genes, are expressed at higher levels in cambium than in differentiating xylem (Zhang *et al.*, 2011). Transcriptome analysis of the poplar cambium annual dormancy–activity transition also shows that a poplar *FIE* homologue is dramatically up-regulated in the dormant phase (Schrader *et al.*, 2004). The poplar FIE protein shows about 90% sequence similarity to the tobacco FIE protein. Thus, it is of interest to investigate the mode of action of the PRC2 complex in the cambial meristem. Moreover, the function of *FIE* homologues in postembryonic axillary meristem activity is largely unknown in vascular plants. Homozygous mutants of the *FIE* gene are embryonic lethal (Ohad *et al.*, 1996), and hypomorphic alleles of this gene cause embryonic flowering resembling *embryonic flower* (*emf*) mutants (Kinoshita *et al.*, 2001). Genomic and epigenomic analyses of the homozygous *fie* mutant that is recovered by a *cdka;1* mutation provide invaluable information about PRC2-mediated gene regulation in the genome-wide view (Bouyer *et al.*, 2011). However, this kind of *fie* homozygous mutant cannot accomplish the transition from embryo to seedling and finally forms callus-like tissue (Bouyer *et al.*, 2011). These characteristics impede the investigations that focus on the role of the *FIE* gene in postembryonic meristem activity in plants.

Virus-induced gene silencing (VIGS) has been developed as a quick and powerful reverse genetics tool to silence endogenous genes in adult plants with the emergence of a variety of plant virus vectors with broad host ranges (Burch-Smith *et al.*, 2004; Becker and Lange, 2010). The vectors based on the tobacco rattle virus (TRV) have been mainly used because of their broad host range and capacity for invading shoot apical meristem (Ratcliff *et al.*, 2001; Liu *et al.*, 2002). In this study, we used the VIGS method to investigate the role of the homologous genes of *FIE* in *Nicotiana benthamiana* in postembryonic shoot development. The silenced plants showed bushy phenotypes and impairment of secondary xylem development. Our results indicated that the *NbFIE* genes regulated auxin homeostasis through repressing the expression of *NbGH3.6*.

## Materials and methods

### Plant materials and growth conditions

*Nicotiana benthamiana* Domin plants were cultivated (one plant per 7 cm×7 cm×7cm pot) in growth chambers (23–26 °C) under a long-day periodicity (16h light–8h dark). Healthy 4-week-old plants with four to five leaves were used for VIGS experiments.

### RNA extraction, RT-PCR and real time qRT-PCR

Fresh plant tissues from independent or pooled biological replicates with the same treatment were ground to fine powder in liquid nitrogen and stored at –80 °C. About 100mg ground material of each sample was used for RNA extraction. To focus on the shoot development and minimize interference from other organs, we only sampled main stems without leaves, petioles and lateral branches for RNA extraction. Total RNA was extracted using the TRIzol Reagent (Invitrogen, Carlsbad, CA, USA) and finally dissolved in 50 μl RNase-free water. The RNA yields varied depending on the sample used. First-strand cDNA was synthesized in a 20 μl reaction system from 2 μg total RNA using the TransScript II One-Step gDNA Removal and cDNA Synthesis SuperMix (TransGen Biotech, Beijing, China) following the manufacturer’s instructions. Twenty microlitres of cDNAs reaction was diluted with 20 μl TE buffer (pH 8.0) and kept at 4 °C until ready for use. For semi-quantitative RT-PCR, 1 μl of diluted cDNA was used in a 20 μl PCR mixture (ExTaq, Takara, Japan or Phusion, NEB, UK) as the starting amount to run 20 cycles in RT-PCR. Twelve microlitres of PCR products were electrophoresed on a 0.8% agarose gel. The amounts of cDNA were adjusted and 20 cycles of PCR were repeated until the internal standard was normalized. Then we performed 30 or 35 cycles of PCR amplification for candidate genes using the calibrated cDNA amounts. For real time qRT-PCR, each target gene was amplified with four technical replicates and 1 μl of diluted cDNA was used as the template in each 20 μl reaction. The relative expression level changes of target genes in qRT-PCR were calculated according to the 2^–ΔΔCT^ method described before (Livak and Schmittgen, 2001).

### Isolation of full-length cDNA of *NbFIE1, NbFIE2* and *NbGH3.6*genes

The full-length cDNA of the putative *NbFIE* genes were amplified using the primers NbFIEfF/NbFIEfR. One microlitre of 20 μl first-strand cDNA reaction product was used as a template for PCR. The PCR products were gel purified and cloned with a pEASY-T1 cloning kit (TransGen Biotech, Beijing, China), and then sequenced to verify their identity as two putative homologues of the *FIE* gene. The sequence alignment was done using Clustal W (http://srs.ebi.ac.uk/) while the phylogenetic tree was constructed using Bioedit software. To clone the *NbGH3.6* full-length cDNA, the 3′-RACE and 5′-RACE reaction was performed with the GeneRacer RACE Ready cDNA kit (Invitrogen, USA). The cDNA from the *NbFIE*-silenced plants acted as the PCR template and the *NbGH3.6* gene-specific primers are listed in Supplementary Table S3 at *JXB* online.

### Gene expression analysis

The procedures for RNA extraction from various plant organs and cDNA preparation were the same as above. Semi-quantitative RT-PCR that used the primers NbFIEfF/NbFIEfR was run under the following programme: 94 °C for 5min, followed by 30 cycles of 94 °C for 30s, 60 °C for 30s, 72 °C for 1min 15s, ended with a 10min 72 °C extension step. The *elongation factor-1 α* (*EF-1α*) homologue in *N. benthamiana* was used as an internal standard (Rotenberg *et al.*, 2006). The two sets of primer pairs for detecting *NbEF1α* are listed in Supplementary Table S2. NbEF1α-realF and NbEF1α-realR were used in the organ expression profile examination of *NbFIE* genes and all real time qRT-PCR reactions. NbEF1α-semiF and NbEF1α-semiR were used in the other RT-PCR experiments. NbGH3.6-realF and NbGH3.6-realR were used in the qRT-PCR reactions for gene expression level detection.

### Virus-induced gene silencing

A cDNA fragment (364-680bp) common in both *NbFIE1* and *NbFIE2* genes was PCR-amplified with the primers NbFIEvF1/NbFIEvR1. The PCR products were cloned into the TRV2 vector (YL156) (Ratcliff *et al.*, 2001) to generate the TRV2-*NbFIE* vector using *Bam*HI and *Xba*I sites. Empty TRV2 vector acted as a control (referred to as negative control plants). These VIGS vectors were introduced into *Agrobacterium* strain GV3101 using the freeze-thaw method (Weigel and Glazebrook, 2006) and VIGS was performed based on the protocol described previously (Burch-Smith *et al.*, 2006).

For gene expression examination, the plant parts above infiltrated leaves, including shoot and leaves from plants with a different treatment, were used for RNA extraction and cDNA biosynthesis as described above. For real time qRT-PCR, NbFIE12RealF and NbFIE12RealR were used to detect both the *NbFIE1* and *NbFIE2* genes. For semi-quantitative RT-PCR examination of *NbFIE1* and *NbFIE2* gene silencing, NbFIEfF and NbFIEfR were used to amplify the full-length cDNA of the *NbFIE1* and *NbFIE2* genes.

### Histological and morphological analysis

The first internodal stem above the cotyledons was longitudinally quadrate-dissected, fixed in formaldehyde (3.7%)–acetic acid (5%)–alcohol (50%) solution, and used for sectioning and maceration experiments. Spurr resin (SPI Chem) or LR white resin (Sigma) was prepared based on the manufacturer’s instructions. Sections (5 μm) were cut on a microtome (Leitz 1512), stained with 1% toluidine blue (Sigma), and photographed under an optical microscope (Zeiss Axioskop 2 plus). To calculate the ratio of sclerenchymatous cells in secondary xylem, sections were photographed under 10×10 magnification, and 15 layers of cells beginning from the outermost cambial cell layer in the visual field were selected. Six independent biological replicates were counted and two quarters of the same first internodal stem were sectioned and counted as technical duplicates. The remaining two quarters of the first internodal stem were used for maceration experiments. Maceration was performed according to Ruzin (1999). For each macerated sample, 20 randomly selected independent visual fields were obtained on several slides. Six independent biological replicates were counted and two quarters of the same first internodal stem were macerated and counted as technical duplicates. For observation of lignin deposition in xylem cell walls, hand-cut sections were stained with phloroglucinol-HCl (1% [w/v] phloroglucinol in 6M HCl) and observed under a dissection microscope.

### RNA-sequencing

Stem tissues of *NbFIE*-silenced and negative control plants excluding leaves, petioles and lateral branches were harvested and frozen in liquid nitrogen at 7 and 10 day after infiltration (DAI), respectively. To avoid the variation between individual plants, 12 independent plants were mixed to prepare each RNA library. Total RNA was extracted using TRIzol® Reagent (Invitrogen) following the manufacturer’s instructions. An Agilent 2100 Bioanalyzer (Agilent Technologies) was employed to analyse RNA concentration and integrity. Library preparation and sequencing reactions were conducted in the Beijing Genome Institute (BGI, Shenzhen, China) (http://www.genomics.cn/index.php) according to the manufacturer’s instructions (Illumina, San Diego, CA, USA). Briefly, poly(A)-containing mRNA was isolated using magnetic beads with oligo(dT) and fragmented into short pieces. Using these short fragments as templates, a random hexamer primer was used to synthesize first-strand cDNAs. Then DNA polymerase I together with buffer, dNTPs, and RNase H were used to synthesize second-strand cDNAs. After purification, end repair, ligation to sequencing adapters and amplification by PCR, the final library was obtained. Finally, the library was sequenced using an Illumina HiSeq™ 2000 platform. After removing adaptor sequences and other unqualified raw reads, clean reads were mapped to *Nicotiana benthamiana* genome scaffold (http://solgenomics.net/) using the SOAP2 software (Li *et al.*, 2009). The SOL (http://solgenomics.net/) and GenBank (http://www.ncbi.nlm.nih.gov/genbank/) databases were employed to annotate the cDNA sequences mapped to the *Nicotiana benthamiana* genome. Differentially expressed genes (DEGs) were identified using the following two criteria based on the method described by Audic and Claverie (1997): (i) absolute fold-change >2 and (ii) *q*-value (false discovery rate (FDR)) <0.05. GO annotations of DEGs were performed to retrieve molecular function, biological process, and cellular component terms using Blast2GO (http://www.blast2go.org/).

### ChIP-PCR

A chromatin immunoprecipiation (ChIP) assay was performed as described by Ricardi *et al.* (2010) with minor modifications. Stem tissues of 12 *NbFIE*-silenced and negative control plants at 10 DAI excluding leaves, petioles and lateral branches were cut as 1cm fragments and then fixed in extraction buffer containing 1% formaldehyde by vacuum infiltration for 10min. The crosslink was stopped by adding glycine and vacuum infiltration was performed for another 5min. Crosslinked tissues were rinsed twice with distilled water, dried thoroughly with paper towels, and finally ground in liquid nitrogen. About 2g tissue powder of each sample was used for nuclei isolation and lysis, DNA shearing and immunoprecipitation, crosslinking reversal and finally DNA recovery following standard steps. Bioruptor (diagenode) was used for DNA shearing and chromatin sonication was done as followed: 10s on–30s off, 20 cycles, cooling on ice for 2min at each five-cycle interval. The anti-trimethyl-histone H3 (Lys27) antibody (Millipore, cat. no. 07-449) was used for immunoprecipitation. ChIPed DNA was resuspended with 50 μl TE solution, and stored at –80 °C. One microlitre of IPed DNA was used as template to do the PCR reaction. The primers used are listed in Supplementary Table S2.

### Free IAA measurements

Stems without leaves and activated axillary buds were collected from gene-silenced and negative control plants at 10 DAI. The material was ground in liquid nitrogen and stored at –80 °C. Measurements of free IAA were conducted in the phytohormonal platform of Institue of Genetics and Developmental Biology, Chinese Academy of Sciences (http://www.genetics.ac.cn/jspt/zwjs/) following the method described by Zhou *et al.* (2010). Approximately 100mg (fresh weight) of plant tissue powder was used for IAA extraction and measurement. Every group had three technical replicates.

Briefly, methanol was used for plant tissue homogenization and extraction with [^2^H]IAA (CDN Isotopes) as an internal standard. Purification of plant extracts was completed with an Oasis Max solid phase extract cartridge (Waters) after centrifugation. The purified hormone-containing fraction was injected into a liquid chromatography–tandem mass spectrometry system composed of an Acquity Ultra Performance Liquid Chromatograph (Acquity UPLC, Waters) and a triple quadruple tandem mass spectrometer (Quattro Premier XE, Waters).

The Genbank accession numbers are *NbFIE1*, JX040473; *NbFIE2*, JX040474; *NbGH3.6*, KP941063.

## Results

### Cloning of the *FIE* homologous genes in *N. benthamiana*

**Fig. 1.**
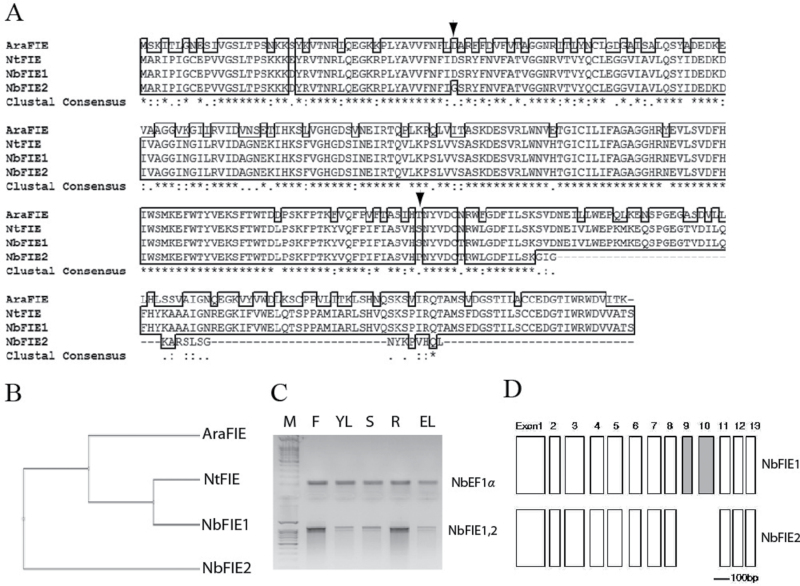
Cloning and expression patterns of *NbFIE1* and *NbFIE2* genes. (A) Sequence alignment and (B) phylogenetic analysis run by the Bioedit program. AraFIE, *Arabidopsis* FIE protein; NtFIE, FIE homologue in *N. tabaccum*. The arrowheads marked the two amino-acid residues in the common coding region of NbFIE1 and NbFIE2. (C) RT-PCR of the organ expression profiles of the *NbFIE1* and *NbFIE2* genes in 7-week-old plants: F, opened flowers; YL, young leaves; S, stem; R, roots; EL, expanded leaves; M, 100-bp DNA ladder marker. The *NbEF1α* gene was used as the internal standard. (D) Schematic depictions of the exon structures of *NbFIE1* and *NbFIE2* genes. Introns are not shown due to uncertainty of sequences and/or size appearing in some of them.

On the basis of the sequence information of *Arabidopsis FIE* (AT3G20740) and the *FIE* homologue in *N. tabaccum* (GenBank: ABY84674.1), two complete coding cDNA fragments were amplified from *N. benthamiana* leaves. The longer cDNA encoded a putative FIE homologue with 74% identity to the *Arabidopsis* FIE protein, while the shorter lacked a 157-bp sequence near its 3′ terminus that caused an open reading frame shift and a truncated C terminus (Fig. 1A, B). Besides the deletion of the 157-bp sequence, the identity of the shorter cDNA differed in 11 nucleic acid bases, and two of them caused alterations of the encoded amino acids (Fig. 1A). We named the longer gene *NbFIE1* and the shorter *NbFIE2*. RT-PCR indicated that both of the *NbFIE* transcripts were present in different organs, with relatively higher levels in opened flowers and roots (Fig. 1C). Recently, the grafted genome sequences were released in the Sol genomic network (SOL, http://solgenomics.net/) (Bombarely *et al.*, 2012). The coding cDNA sequences of *NbFIE1* and *NbFIE2* were used respectively for BLAST in SOL. The BLAST results showed that the lack of two exons in *NbFIE2* gene locus caused its coding cDNA sequence to be 157bp shorter than *NbFIE1* (Fig. 1D).

### VIGS of *NbFIE* genes

**Fig. 2.**
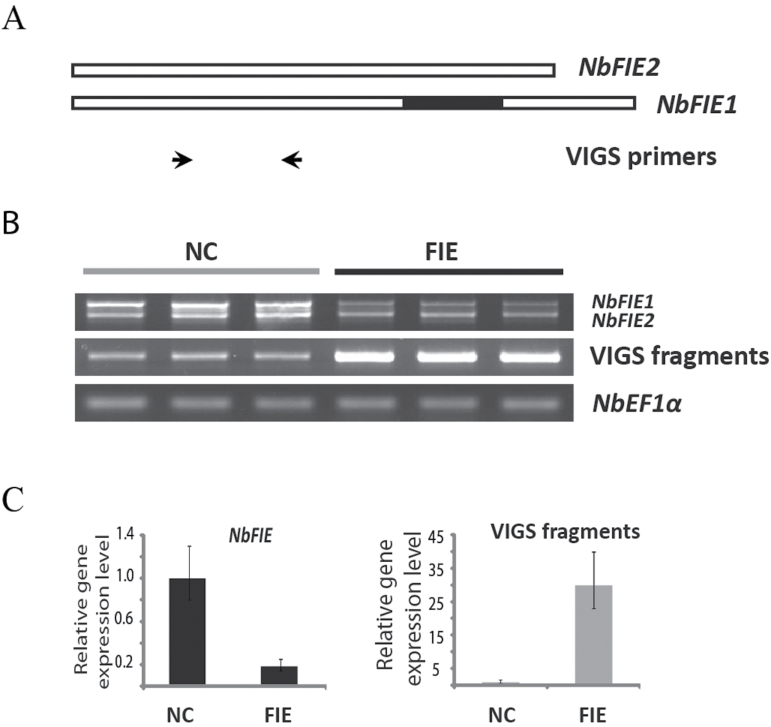
VIGS experiments of *NbFIE* genes. (A) Schematic depiction of the structures of the *NbFIE1* and *NbFIE2* coding sequences and the positions of the primers of VIGS used. The black bar represented the specific 157bp cDNA sequence of *NbFIE1*. The arrowheads marked the positions of the primers. (B) Semi-quantitative RT-PCR of *NbFIE* genes and VIGS fragments in control plants and *NbFIE* silenced plants at 21 days after infiltration (DAI). (C) Real-time qRT-PCR of *NbFIE* genes and VIGS fragments in control plants and *NbFIE* silenced plants at 21 DAI. Plant shoot and leaf tissues above infiltrated leaves were used for RNA extraction and RT-PCR. The *NbEF1α* gene was used as the internal standard. NC, control; FIE, *NbFIE* silenced plants. Error bars indicate standard deviation calculated from three technical repeats.

**Fig. 3.**
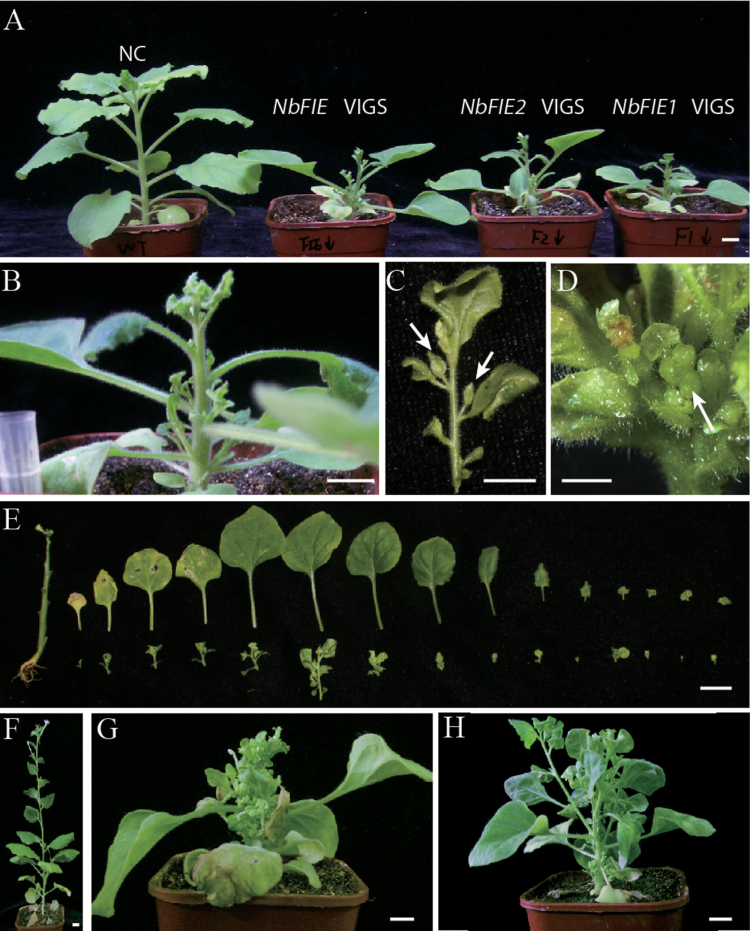
VIGS experiments to silence both *NbFIE1* and *NbFIE2* genes. (A) Plants at 24 DAI from VIGS experiments using the common fragment of *NbFIE1* and *NbFIE2* (*NbFIE* VIGS) as well as the full-length cDNA of the *NbFIE1* gene (*NbFIE1* VIGS) or the *NbFIE2* gene (*NbFIE2* VIGS). NC, control plants. (B) A side view of an *NbFIE*-silenced plant at 24 DAI. (C) A typical first-order lateral branch from an *NbFIE*-silenced plant at 24 DAI. The arrows showed the extended second lateral branches. (D) A magnified view of the inflorescence meristem on an *NbFIE*-silenced plant at 24 DAI. The arrow indicates that the inflorescence meristem arrested with several tiny organs. (E) A dissected *NbFIE*-silenced plant at 24 DAI. Each of first-order axillary buds and lateral branches (the lower line) paralleled the corresponding nodal leaves (the upper line). (F) A negative control plant at 56 DAI. (G, H) *NbFIE*-silenced plants at 56 DAI with strong and mild phenotypes. Bars in (A–C) and (E–H), 1cm; bar in (D), 0.5cm.

We cloned a coding fragment (364–680bp) common to *NbFIE1* and *NbFIE2* as well as their full-length cDNAs respectively into TRV vectors to silence both *NbFIE1* and *NbFIE2* genes (Fig. 2A). Results of both RT-PCR and real-time qRT-PCR indicated that in silenced plants using the partial cDNA fragment the expression of endogenous *NbFIE1* and *NbFIE2* genes was downregulated significantly, while the transcripts of VIGS fragments dramatically increased (Fig. 2B, C). All the VIGS experiments with these three vectors gave the same results (Fig. 3A). Therefore, we followed the TRV vectors bearing the common fragment of *NbFIE1* and *NbFIE2* for further analyses of phenotype and gene expression. At least 10 different batches of VIGS treatments (about 500 plants) were performed. Almost all the infiltrated plants showed similar phenotypes. For simplicity, we used the term ‘*NbFIE*-silenced’ to indicate the plants silenced for both *NbFIE1* and *NbFIE2*.

### Phenotypes of the *NbFIE*-silenced plants

The *NbFIE*-silenced plants at 24 day after infiltration (DAI) showed dramatically altered developmental events. Numerous outgrowths of first-order axillary buds altered the architecture of the silenced plants (Fig. 3B–E). In addition, most of second axillary buds were activated on first-order lateral branches of *NbFIE*-silenced plants (Fig. 3C). During the time course of gene silencing, newly emerged leaves showed gradually transformed shapes from hyponastic to tiny irregular lobed, which were considered a sensitive visual indicator of the spread of silencing (Fig. 3E). The activity of inflorescence meristems gradually terminated, leaving only several aborted tiny organs clustered on the shoot tip at 24 DAI (Fig. 3E). In negative control plants at 56 DAI, there were seldom first-order lateral branches (Fig. 3F) while in *NbFIE*-silenced plants, nearly all axillary buds were activated and most of them extended to form obvious first-order lateral branches, which made the silenced plants bushy (Fig. 3G, H).

**Fig. 4.**
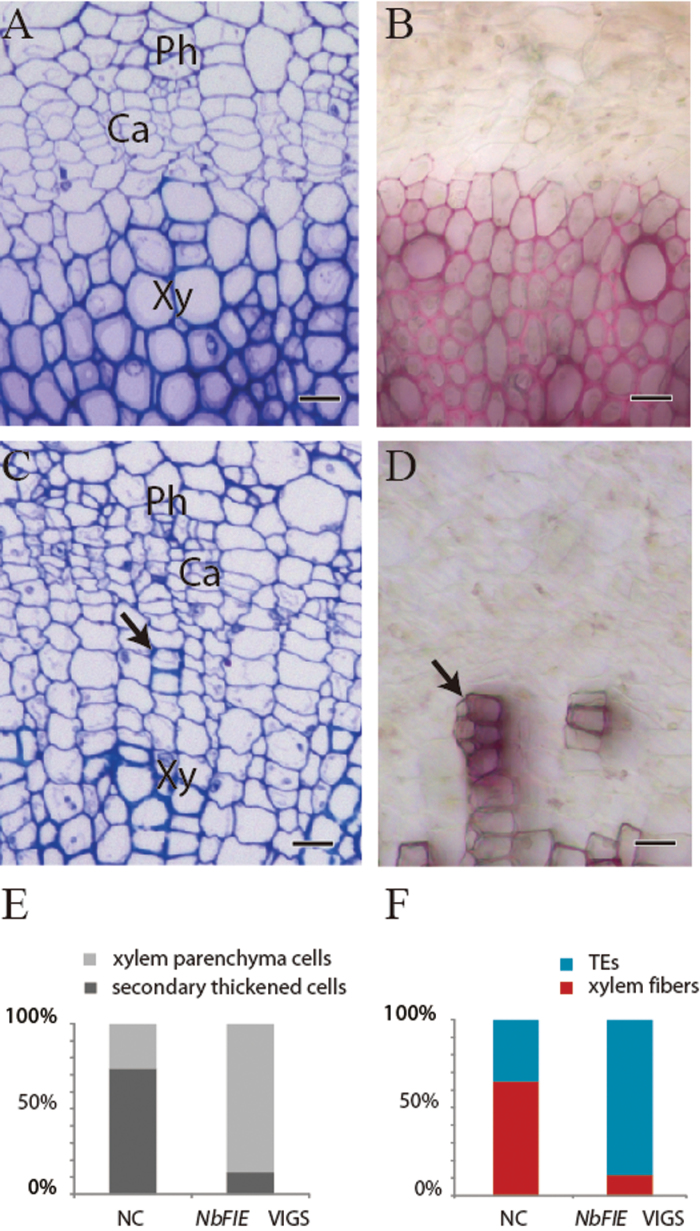
Anatomical analyses of secondary xylem differentiation. Cross sections of the first internode above the cotyledons in a control plant (A, B) and in an *NbFIE*-silenced plant (C, D) at 24 DAI. (A, C) plastic sections stained by Toluidine blue O. (B, D) Hand cross sections stained with Phloroglucinol-HCl. The arrow in (C) indicates dispersed secondary thickened cells. The arrow in (D) indicates dispersed lignified cells. (E) Ratios of secondarily thickened cells to xylem parenchyma cells. (F) Ratios of tracheary elements to xylem fibres in the population of secondarily thickened cells. NC, negative control plants; *NbFIE* VIGS, *NbFIE*-silenced plants. Bars in (A–D), 50 μm.

To determine the role of *NbFIE* genes in shoot development, we investigated the anatomical changes in the first internodes above the cotyledons of differently treated plants at 24 DAI. Compared with negative controls (Fig. 4A), secondary xylem cells in *NbFIE*-silenced plants rarely formed secondary cell walls (Fig. 4C), which indicated that the differentiation into xylem cells was greatly suppressed. Phloroglucinol-HCl staining of hand-cut sections at the same sampling location showed much weaker lignin staining in the secondary xylem of the *NbFIE*-silenced plants (Fig. 4D) than control plants (Fig. 4B). The statistical data of cell numbers from cross-sections confirmed that the ratio of the xylem cells with secondary walls to cambial and xylem parenchyma cells was remarkably reduced from 73% (in the negative controls) to 12% (in the *NbFIE*-silenced plants) (Fig. 4E and see Materials and methods). The results of maceration experiments of the first internodes above the cotyledons showed that in the population of the xylem cells with secondary walls, the ratio of xylem fibres to tracheary elements was also notably reduced, which indicated that the differentiation of xylem fibres was largely retarded (Fig. 4F and see Materials and methods).

### RNA Sequencing analysis of *NbFIE*-silenced plants to identify putative target genes of the PRC2 complex involved in shoot development

Based on the fact that PRC2 complexes act as transcription repressors, we speculated that the pleiotropic effect of *NbFIE* deficiency on plant development was due to the de-repression of certain target genes. To reveal a wider range of genomic programmes regulated by *NbFIE* genes, we compared the transcriptional profiles in the stem of *NbFIE*-silenced and control plants using high-throughput RNA sequencing technology.

**Fig. 5.**
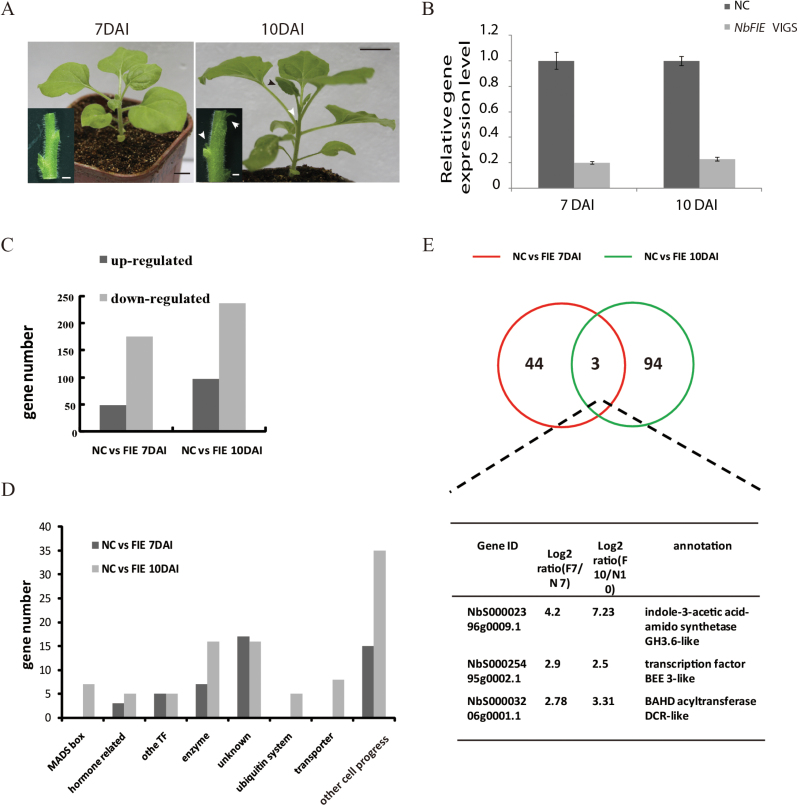
RNA-seq analysis at 7 and 10 DAI. (A) Representative *NbFIE*-silenced plant at 7 and 10 DAI. Inlets, dissected stems to show axillary buds. White arrowheads indicated sprouted axillary buds; the black arrowhead showed the wrinkle of leaf margin. (B) The expression level of *NbFIE* genes in *NbFIE*-silenced plants at 7 and 10 DAI. Error bars indicate standard deviation calculated from three technical repeats. (C) The number of up-regulated and down-regulated genes (log2 ratio>2, and *P*-value<0.01) in *NbFIE*-silenced genes at 7 and 10 DAI. (D) Function categories of up-regulated genes in *NbFIE*-silenced genes at 7 and 10 DAI. (E) Overlapped up-regulated genes at 7 and 10 DAI. Bars (black) in (A), 1cm. Bars (white) in inlets of (A), 1mm.

We first checked the time course of gene expression and phenotypes. At 7 DAI, there were no distinguishable phenotypes observed (Fig. 5A), but RT-PCR examination showed that endogenous *NbFIE* genes were already down-regulated (Fig. 5B). While at 10 DAI, the newly formed leaves of *NbFIE*-silenced plants were slightly wrinkled and tiny first axillary buds began to sprout (Fig. 5A).

Since the axillary bud outgrowth was observed at about 10 DAI, the activation of downstream target genes related to the shoot branching must happen before the appearance of axillary buds. Thus, we compared the gene expression profiles using *NbFIE*-silenced and negative control plants at 7 and 10 DAI. To focus on the shoot branching as well as secondary vascular development, and minimize interference from other organs, we only sampled main stems without leaves, petioles and lateral branches for RNA extraction. Subsequently, the resulting cDNA from the four samples was sequenced using Illumina technology. The sequences obtained were then aligned to *Nicotiana benthamiana* cDNA database using the SOAP2 software (see Materials and methods). The SOL, NCBI and GenBank databases were employed to annotate the cDNA sequences mapped to the *Nicotiana benthamiana* genome.

The genes with activated mRNA accumulation in *NbFIE*-silenced plants at 7 and 10 DAI were identified by screening for cDNAs that had >2.0-fold more relative expression levels than those in control plants. Only the genes having the expression level change with a *P*-value <0.01 were initially considered (see Materials and methods). We identified dozens of genes that exhibited reduced expression levels in *NbFIE*-silenced plants, and unexpectedly, the down-regulated genes at both time points were markedly more abundant than the up-regulated ones (Fig. 5C). However, those down-regulated genes were not studied in detail in this report because we were hunting for direct target genes of PRC2, which were supposed to be activated in *NbFIE*-silenced plants. Finally, a total of 47 and 97 up-regulated genes were identified at 7 DAI and 10 DAI, respectively, shown in Supplementary Tables S1 and S2.

A gene ontology (GO) analysis was performed on the 47 and 97 up-regulated genes, and we found several target candidate genes. Homologue genes of *AGAMOUS* (*AG*) and *FLOWERING LOCUS T* (*FT*), which are known to be target genes of PRC2 in *A. thaliana* (Moon *et al.*, 2003; Katz *et al.*, 2004; Jiang *et al.*, 2008), were highly up-regulated in 10 DAI *NbFIE*-silenced plants. Other *AGAMOUS*-like MADS box genes also displayed overt de-repression in 10 DAI silenced plants (see Supplementary Table S1). Besides MADS box genes, other transcription factor genes like a homologue of *ATMYB48*, which regulates flavonoid pathways (Xu *et al.*, 2015), were de-repressed in 10 DAI *NbFIE*-silenced plants. Correspondingly, a flavonoid biosynthesis oxidoreductase gene was also up-regulated. The upregulation of 11 genes including arabidopsis transcription factor homologue genes and their related genes was confirmed by qRT-PCR (Supplementary Fig. S1). In addition, enzymes involved in various cellular processes were over-represented. A few genes participating in the ubiquitin system and nutrient transport were only enriched in 10 DAI *NbFIE*-silenced plants, suggesting these cell processes may occur as the later response of *NbFIE* gene silencing (Fig. 5D).

Considering the fact that axillary bud outgrowth was observed at about 10 DAI in *NbFIE*-silenced plants, the related target gene should be activated earlier than 10 DAI and still maintain high enrichment later, so we checked those genes with high expression levels at both 7 and 10 DAI. Surprisingly, there were only three candidate genes up-regulated at both time points, which are listed in Fig. 5E. One of these three genes, *NbS00002396g0009.1*, was a homologue of *GH3.6* in *Arabidopsis*, which is responsible for maintaining auxin homeostasis through conjugating excess IAA to amino acids. This gene was chosen because it exhibited the most dramatic expression level change at both time points, 4.2 and 7.23-fold (log2 ratio), respectively, and it appeared to have a close relationship with the auxin signal pathway, which is undoubtedly related to shoot branching and xylem differentiation.

### *NbGH3.6* was a direct target of the PRC2 complex in *N. benthamiana*

**Fig. 6.**
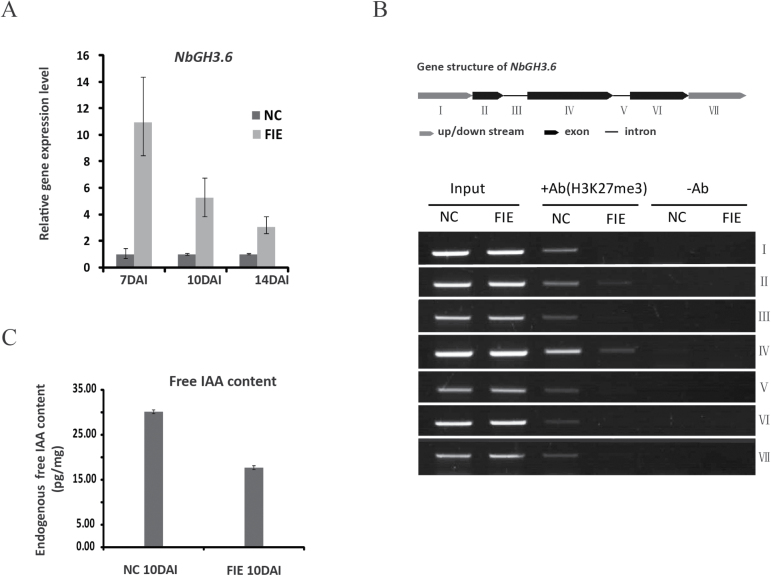
Expression analysis, ChIP assays of *NbGH3.6*, and free IAA measurements. (A) Real-time qRT-PCR of the *NbGH3.6* gene at 7, 10 and 14 DAI. Error bars indicate standard deviation calculated from three technical repeats. (B) ChIP assays of NbGH3.6 in control plants and *NbFIE*-silenced plants at 10 DAI. +Ab, added antibodies; –Ab, without antibodies. (C) Free IAA measurements of negative control plants and *NbFIE*-silenced plants at 10 DAI. NC, control plants; FIE, *NbFIE*-silenced plants. Standard errors were calculated from three technical repeats.

We next cloned the full-length cDNA sequence of *NbS00002396g0009.1*. We performed 3′- and 5′-RACE to get the correct cDNA ends, and named it *NbGH3.6*, because the protein sequence alignment showed high similarity (74%) with Arabidopsis GH3.6. The relative expression level of *NbGH3.6* was further examined by qRT-PCR, and the results indicated that *NbGH3.6* was continuously activated in *NbFIE*-silenced plants from 7 to 14 DAI (Fig. 6A), which coincides with the expected expression pattern of PRC2 target genes.

To investigate whether *NbGH3.6* was a direct target of PRC2 or not, we performed a ChIP assay and detected the H3K27me3 marks on the *NbGH3.6* locus. Using a ChIP-reliable antibody that was specifically against H3K27me3, we immunoprecipitated the enriched DNA sequences with H3K27me3 marks in *NbFIE*-silenced and control plants at 10 DAI. As expected, we found strong enrichment for sequences from multiple sites within the *NbGH3.6* locus in control plants, while their enrichment bands were below detectable levels in *NbFIE*-silenced plants (Fig. 6B). The ChIP results indicated that H3K27me3 marks were dispersed over multiple sites on the *NbGH3.6* locus, and suggested that the activation of *NbGH3.6* gene in *NbFIE*-silenced plants may be caused by the lack of repressive histone methylation marks (H3K27me3).

### The free endogenous IAA content was reduced in *NbFIE*-silenced plants

Given the fact that *GH3.6* plays an important role in maintaining auxin homeostasis by conjugating excess IAA to amino acids (Staswick *et al.*, 2005), the activation of *NbGH3.6* could lead to the decrease of free IAA in *NbFIE*-silenced plants. To confirm that, we measured the free endogenous IAA content in stems of both *NbFIE*-silenced and control plants at 10 DAI. The results indicated a significant decline of free IAA content by nearly 50% in *NbFIE*-silenced plants (Fig. 6C). This result indicates that the down-regulation of *NbFIE* eventually leads to IAA deficiency.

## Discussion

### PRC2 function in axillary meristem and cambium activity

In recent years, great progress has been made in the understanding of the PRC2 complex, a key regulator of epigenetic states catalysing catalysing the trimethylation of histone H3 at lysine 27 in Arabidopsis (Hennig and Derkacheva, 2009). Besides the fundamental roles of repressing gene expression through H3K27 trimethylation, multiple mutations of PRC2 also demonstrated that PRC2 is crucial in several plant developmental programmes: (i) endosperm formation in flowering plants (Chaudhury *et al.*, 1997); (ii) major phase transition, e.g. from vegetative growth to flowering and embryo to seedling transition (Kinoshita *et al.*, 2001; Katz *et al.*, 2004); and (iii) maintaining overall cell and tissue organization (Bouyer *et al.*, 2011). However, in all the Arabidopsis mutations, the PRC2 function is deficient in initial embryo formation and subsequent organ differentiations, which make it a great challenge to investigate the roles of PRC2 in postembryonic development, such as the axillary meristem and secondary vascular development. Thus, whether and how PRC2 is essential for the postembryonic development programme once the correct organ initiations have been established remained an open question. Here we used the VIGS technology to investigate PRC2 functions in 4-week tobacco plants, and the results showed that a decline of endogenous *NbFIE1* and *NbFIE2* transcripts caused a stunted and bushy shape through the overgrowth of axillary buds, and meanwhile, the differentiation into xylem cells from the cambium was greatly suppressed. We supposed that PRC2 deposited correct epigenetic repression marks on chromatin in the daughter cells of meristem to assure the correct gene expression level, so that a certain set of gene families involved in maintaining apical dominance and cell division activity must be de-repressed once *FIE* genes were down-regulated in *N. benthamiana*. Such mechanisms might be broadly conserved in other plant species. For example, in Arabidopsis, lines with suppression of the *FIE* gene also have short internodes and increased number of rosette inflorescence stems (Katz *et al.*, 2004). In *P. patens*, knockout of the native *PpFIE* gene also leads to over-proliferation of apical cells, which function as meristematic cells, and finally to formation of a cluster of aberrant developed buds on the apex (Mosquna *et al.*, 2009). These results strongly suggested that PRC2 function is required in the whole life of plants to maintain the accurate meristem activity by modulating gene expression levels.

### PRC2 regulates the auxin pathway in plants

*GH3* family genes contribute to auxin homeostasis by conjugation of excess IAA to amino acids in both Arabidopsis and rice (Nakazawa *et al.*, 2001; Staswick *et al.*, 2005; Zhang *et al.*, 2009). High expression level of *GH3* genes could lead to the deficiency of endogenous free IAA (Nakazawa *et al.*, 2001; Staswick *et al.*, 2005). Overexpression of the *iaaL* gene from *Pseudomonas savastanoi* in *Nicotiana tabacum*, which encoded an IAA-lysine synthetase, resulted in reduced IAA levels, wrinkled leaves, reduced apical dominance and inhibition of vascular differentiation in transgenic plants (Romano *et al.*, 1991). These phenotypes are quite similar to the ones of our *NbFIE*-silenced plants. Interestingly, here we found that *NbGH3.6* was significantly activated in *NbFIE*-silenced plants, and the endogenous free IAA content showed a strong reduction in *NbFIE*-silenced plants, possibly as the result of high expressiom of *NbGH3.6.* Moreover, ChIP-PCR indicated that *NbGH3.6* was a direct target of the PRC2 complex. Considering the vital roles of auxin in repressing axillary bud outgrowth and cambial cell differentiation, the phenotypes that *NbFIE*-silenced plants displayed can possibly be explained by activation of *NbGH3.6* expression and consequent reduced endogenous free IAA content.

Although whether PRC2 is involved in regulation of phytohormonal signal pathways remains unclear currently, more and more evidence shows that there is a connection between the PRC2 complex and auxin signal pathway (Li *et al.*, 2008; Lafos *et al.*, 2011; He *et al.*, 2012; Xiao and Wagner, 2015). A whole-genome tiling array for identifying the H3K27me3 targets in undifferentiated shoot apical meristem cells and differentiated leaf cells indicated H3K27me3 marks deposited on genes involved in the auxin signal pathway, including biosynthesis, perception, polar transport, and signal transduction (Lafos *et al.*, 2011). In addition, ChIP-chip data revealed that H3K27me3 levels of auxin-pathway genes including several auxin-inducible genes, such as *GH3* genes and *AUX/IAA* genes, are dramatically reduced in the callus compared with the leaf (He *et al.*, 2012). These results and our data strongly suggest that PRC2 functions in auxin homeostasis and signaling. *GH3* genes are auxin early responsive genes (Terol *et al.*, 2006). Therefore the epigenetic regulation of *NbGH3.6* by the *NbFIE* genes implies that PRC2 is also involved in auxin responsiveness.

In summary, our study showed that *NbFIEs* play an important role in plant postembryonic shoot development. *NbFIEs* repress the expression of *NbGH3.6* and regulate the axillary bud growth and secondary xylem differentiation through tuning endogenous auxin homeostasis in *Nicotiana benthamiana*. Thus our study has added insight about the function of PRC2 in plant development.

## Supplementary data

Supplementary data are available at *JXB* online.

Supplementary Table S1. Gene expression profiles of negative control and NbFIE-silenced plants at 7 DAI.

Supplementary Table S2. Gene expression profiles of negative control and NbFIE-silenced plants at 10 DAI.

Supplementary Table S3. All the primers used in this study.

Supplementary Figure 1. qRT-PCR confirmation of the up-regulation of homologues of MADS box gene, *MYB48*, *FT* and their related genes that were revealed by RNA-seq in *NbFIE*-silenced plants at 10 DAI.

Supplementary Data
