# Supplementary material for: Fertilization Independent Endosperm genes repress NbGH3.6 and regulate the auxin level during shoot development in Nicotiana benthamiana
Source: J Exp Bot. 2016 Feb 11;67(8):2207–17. doi: 10.1093/jxb/erw024 (PMC4809283; doi:10.1093/jxb/erw024)
Supplement: Supplementary Data [file supp_67_8_2207__index.html]

 Fertilization Independent Endosperm genes repress NbGH3.6 and regulate the auxin level during shoot development in Nicotiana benthamiana — Fertilization Independent Endosperm genes repress NbGH3.6 and regulate the auxin level during shoot development in Nicotiana benthamiana — Supplementary Data 

# *Fertilization Independent Endosperm* genes repress *NbGH3.6* and regulate the auxin level during shoot development in *Nicotiana benthamiana*

## Supplementary Data

Data files

- supplementary\_figure\_S1.pdf - Supplementary Data
- supplementary\_table\_S1.xls - Supplementary Data
- supplementary\_table\_S2.xls - Supplementary Data
- supplementary\_table\_S3.xlsx - Supplementary Data
