# Supplementary material for: Fertilization Independent Endosperm genes repress NbGH3.6 and regulate the auxin level during shoot development in Nicotiana benthamiana
Source: J Exp Bot. 2016 Feb 11;67(8):2207–17. doi: 10.1093/jxb/erw024 (PMC4809283; doi:10.1093/jxb/erw024)
Supplement: Supplementary Data [file supp_erw024_supplementary_figure_S1.pdf]

a

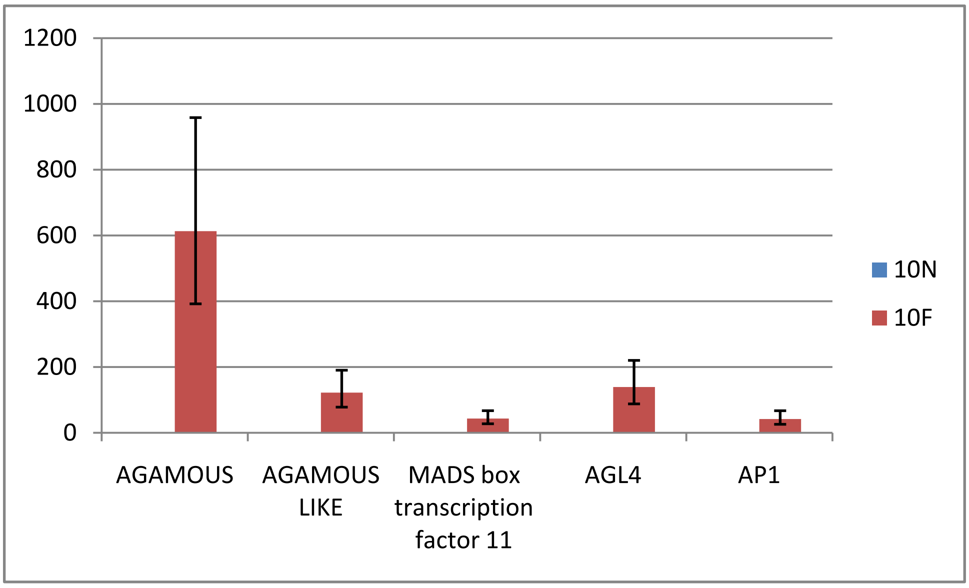

b

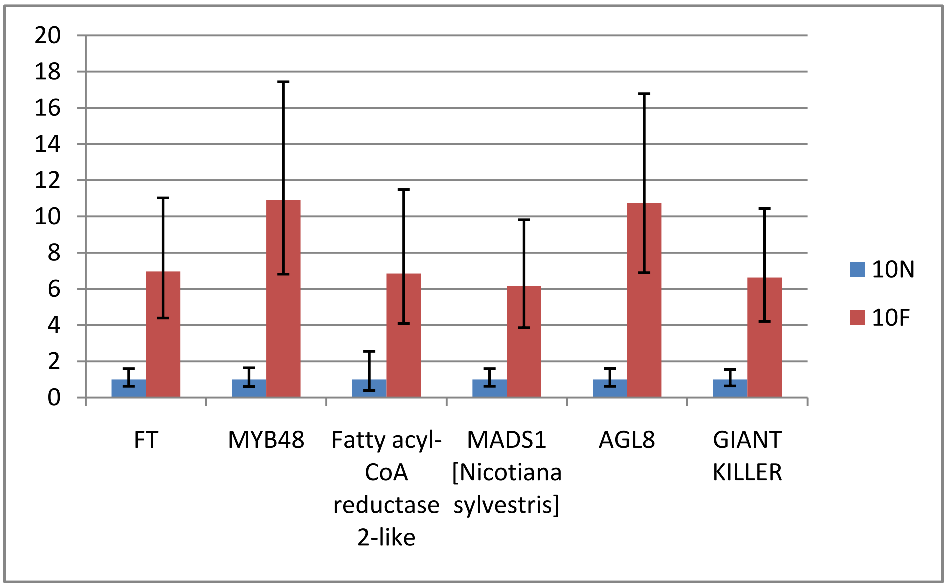

Figure S1. qRT-PCR confirmation of the upregulation of homologs of MADS box gene, *MYB48*, *FT* and their related genes that were revealed by RNA-seq in *NbFIE*-silenced plants at 10 DAI. (a) MADS box genes with highly de-repression. (b) MADS box genes with mild release and other genes. *GIANT KILLER* is a direct target of AGAMOUS in *Arabidopsis*. 10N, negative control plants; 10F, *NbFIE*-silenced plants.
